# Redox Conformation-Specific Protein–Protein Interactions of the 2-Cysteine Peroxiredoxin in Arabidopsis

**DOI:** 10.3390/antiox9060515

**Published:** 2020-06-11

**Authors:** Michael Liebthal, Johannes Schuetze, Anna Dreyer, Hans-Peter Mock, Karl-Josef Dietz

**Affiliations:** 1 Department of Biochemistry and Physiology of Plants, Faculty of Biology, University of Bielefeld, 33615 Bielefeld, Germany; mliebthal@uni-bielefeld.de (M.L.); anna.dreyer@uni-bielefeld.de (A.D.); 2 Angewandte Biochemie, Leibniz-Institut für Pflanzengenetik und Kulturpflanzenforschung (IPK), Corrensstraße 3, D-06466 Seeland, Germany; j.schuetze@gmx.net (J.S.); mock@ipk-gatersleben.de (H.-P.M.)

**Keywords:** 2-cysteine peroxiredoxin, interactome, thioredoxin, proteomics, redox, thiol

## Abstract

2-Cysteine peroxiredoxins (2-CysPRX) are highly abundant thiol peroxidases in chloroplasts and play key roles in reactive oxygen species (ROS) defense and redox signaling. Peroxide-dependent oxidation of cysteines induces conformational changes that alter the ability for protein–protein interactions. For regeneration, 2-CysPRXs withdraw electrons from thioredoxins (TRXs) and participate in redox-dependent regulation by affecting the redox state of TRX-dependent targets, for example, in chloroplast metabolism. This work explores the redox conformation-specific 2-CysPRX interactome using an affinity-based pull down with recombinant variants arrested in specific quaternary conformations. This allowed us to address a critical and poorly explored aspect of the redox-regulatory network and showed that the interaction of TRXs, their interaction partners, and 2-CysPRX occur under contrasting redox conditions. A set of 178 chloroplast proteins were identified from leaf proteins and included proteins with functions in photosynthesis, carbohydrate, fatty acid and amino acid metabolism, and defense. These processes are known to be deregulated in plants devoid of 2-CysPRX. Selected enzymes like LIPOXYGENASE 2, CHLOROPLAST PROTEIN 12-1, CHORISMATE SYNTHASE, ß-CARBONIC ANHYDRASE, and FERREDOXIN-dependent GLUTAMATE SYNTHASE 1 were subjected to far Western, isothermal titration calorimetry, and enzyme assays for validation. The pull down fractions frequently contained TRXs as well as their target proteins, for example, FRUCTOSE-1,6-BISPHOSPHATASE and MALATE DEHYDROGENASE. The difference between TRX-dependent indirect interactions of TRX targets and 2-CysPRX and direct 2-CysPRX binding is hypothesized to be related to quaternary structure formation, where 2-CysPRX oligomers function as scaffold for complex formation, whereas TRX oxidase activity of 2-CysPRX controls the redox state of TRX-related enzyme activity.

## 1. Introduction

Plants tune their metabolism and energy conversion in dependence on environmental conditions. Posttranslational modifications (PTMs) and controlled changes in protein–protein interactions play decisive roles in this regulation. Thiol PTMs are evolutionarily ancient mechanisms employed in all organisms for regulation of diverse cellular processes. They are especially elaborate in photosynthetic chloroplasts harboring the photosynthetic electron transport (PET) [1]. The chloroplast thiol-redox regulatory network consists of specific components. Redox input elements like ferredoxin (FDX), NADPH, and glutathione feed electrons into the network. Redox transmitters like thioredoxins (TRXs) and TRX-like proteins transfer electrons to the redox target proteins. Reactive oxygen species (ROS) function as final electron acceptors. They preferentially react with redox sensors such as peroxiredoxins (PRXs), which withdraw electrons from redox transmitters [2,3]. Electron flow follows gradients in redox midpoint potentials from around −440 mV in the case of FDX to positive values of ROS such as H_2_O_2_, but redox midpoint potentials of most regulatory thiols range between −330 mV and −260 mV [4].

PRXs (EC 1.11.1.15) decompose peroxides by reaction with their peroxidatic thiol located in a conserved catalytic environment. Most PRXs prefer H_2_O_2_ as substrate, but also accept alkyl hydroperoxides and peroxynitrite [5]. After their first identification in yeast (*Saccharomyces*, [6]) and bacteria (*Salmonella* [7]), PRXs were found in mammals (*Rattus* [8]) and plants (*Hordeum*, [9]). Initial experiments characterized PRX as protector proteins of nucleic acids and proteins, and later as thiol peroxidases and chaperones and interactors.

Plant genomes encode four of six different PRX subgroups, namely, 1-CysPRX, typical 2-CysPRX, atypical PRXII, and PRXQ. They are found in all organs, cells, and many subcellular compartments, namely in the nucleoplasm and cytosol of specific cells such as the aleuron layer of seeds (1-CysPRX), as well as in the cytosol (PRXIIB/C/D), plastids (2-CysPRX, PRXIIE, PRXQ), and mitochondria (PRXIIF) [10]. 2-CysPRX is the most abundant plant PRX, ranking at the 14th and 16th position of chloroplast proteins in *Arabidopsis* [11] with an estimated total concentration of 100 µM in the stroma.

2-CysPRX functions as thiol peroxidase and, as shown recently, as TRX oxidase in regulation of the Calvin–Benson cycle (CBC) and malate dehydrogenase activity in changing light conditions similar to PRXQ [12,13]. In the thiol peroxidase cycle, the peroxidatic cysteine (Cys_P_) reduces the peroxide, and its thiol oxidizes to the sulfenyl derivative. The sulfenyl residue converts to an intermolecular disulfide with the resolving thiol (Cys_R_) and water is released. Prior to the next peroxidase cycle, redox transmitters such as TRXs reduce the disulfide bond to the thiol form [10].

ROS-dependent oxidation of the sulfenyl group to sulfinyl or sulfonyl derivatives occurs as a side reaction with an average propensity relative to peroxidase cycle of 1:250 [14], and leads to a hyperoxidized form. Sulfiredoxins (SRX) retro-reduce the sulfenyl group to the active thiol with very slow turnover rate [15]. The hyperoxidized 2-CysPRX adopts a high molecular weight conformation with chaperone function. Thus, in addition to peroxidase function protein–protein interactions define a second important function of 2-CysPRX as chaperone, binding partner, circadian read-out, and redox sensor [3,10,16]. Importantly, all functions are linked to the five redox-dependent conformations, namely, reduced dimer, reduced decamer, oxidized dimer, hyperoxidized decamer, and hyperoxidized hyperaggregates [17].

This work aimed to address the role of protein–protein interactions for the physiological function of chloroplast 2-CysPRX. A previous interactome study exploited co-pull down with anti-2-CysPRX antiserum from wildtype and *2-cysprxAB* knockout plants without distinguishing conformation and redox state [16]. As pointed out above, 2-CysPRX adopts five different redox-dependent conformations. This complexity could not be controlled in that study. In addition, the interacting proteins may also adopt different redox states. 

Therefore, this work makes use of the site-directed mutated variants of Arabidopsis 2-CysPRX introduced by König et al. [14], namely wildtype (WT) and the pseudoreduced C54S and the pseudohyperoxidized C54D variant. A serine substitutes the peroxidatic cysteine in C54S mimicking the reduced conformation even in oxidizing buffer. Aspartate instead of Cys54 introduces a negative charge and a more bulky side group and mimics the hyperoxidized sulfinylated form. Binding to recombinant immobilized protein, a stepwise elution, mass spectrometric identification, and selected validation were sought to elucidate the complex interactome of 2-CysPRX.

## 2. Materials and Methods

### 2.1. Plant Growth and Extraction of Proteins

*Arabidopsis thaliana* wild type (Col-0) plants were grown at 100 µmol photons m^−2^s^−1^ in short day (10 h light). Temperature was set to 21 °C during the day and 18 °C at night with 50% relative humidity. Plants grown for six weeks were used to isolate leaf proteins. Leaves were extracted in buffer A (50 mM 2-[4-(2-hydroxyethyl)piperazin-1-yl]ethanesulfonic acid [HEPES], pH 8.0) supplemented with either 1 mM H_2_O_2_ or 10 mM dithiothreitol (DTT). Crude extract was filtered twice through four layers of cloth, cleared by centrifugation, and stored on ice (4 °C). Protein concentration was determined using the Bradford assay calibrated with bovine serum albumin (BSA).

### 2.2. Affinity-Based Interaction Assay

Recombinant hisx6-tagged 2-CysPRXA was pretreated for 30 min in buffer A (50 mM HEPES, pH 8.0) with either 1 mM H_2_O_2_ or 10 mM DTT for redox adjustment. Each column was filled with 2 mL Ni-NTA-agarose (1 mL effective beads) and bound with 3 mg recombinant protein using buffer A (50 mM HEPES, pH 8.0), followed by intensive washing. Leaf protein pretreated with 10 mM DTT or 1 mM H_2_O_2_ during extraction was incubated on columns for 30 min in separate sets for reducing and oxidizing conditions, namely with 4 (3 variants plus control) × 2 (conditions) columns. As unspecific binding control, a column was loaded with Ni-nitrilotriacetic acid (NTA)-agarose treated exactly the same way, but without recombinant protein. After 30 min of redox adjustment, columns were washed with buffer A. Plant extract (50 mg protein) was added to the columns and incubated with slow shaking for 1 h at 4 °C. Only reduced extract was combined with reduced 2-CysPRX, and oxidized extract with oxidized 2-CysPRX. Unbound protein was washed off with 30 mL buffer A. Stepwise elution was realized with 1 mL volumes of (I) high salt 1 M NaCl, (II) 10 mM DTT for reduction of previously oxidized columns, and (III) 250 mM imidazole, dissolved in buffer A. Columns were washed with 30 mL buffer A between each elution step.

### 2.3. Peptide Preparation and Mass Spectrometric Analysis

Proteins from each elution were precipitated, quantified (2-D Quant Kit, GE Healthcare), and concentrated by ethanol precipitation if necessary, before MS analysis. Digestion with trypsin was done overnight using the filter-aided sample preparation (FASP) for each 40 µL sample (*n* = 3 experiments × 2 technical replicates per elution condition). Peptides were separated on a 15 cm PepMap 100 C18 column (Thermo Fisher) using a 60 min gradient and detected by mass spectrometry (MS) with an Impact II instrument (Bruker Daltonik, Bremen, Germany). Raw data were analyzed, and peptides were identified against the UniRef90 database. Positive assignments required the following criteria: (I) minimum of two independent peptides per protein, (II) peptides with Mascot Score of 25 or higher, (III) peptides found in at least 2 out of 3 samples or 4 out of 6 MS runs, and (IV) peptides not detected in the sample from the control column without 2-CysPRX.

### 2.4. Cloning and Heterologous Expression of Recombinant Proteins

CHORISMATE SYNTHASE (CS) was cloned into the pET28a vector. Primers used for cloning were the following: fwd ATC CAT GGA TGA CTG GAA GTT CAT ATG GG and rev TTG AGC TCC AAA GCA GTA GCA TTT TGC, LIPOXYGENASE 2 (LOX2: fwd CCC GCT AGC ATG GCA CAG AAT ATT AAA GTA, rev CCC CGG ATC CTC AAA TAG AAA TAC TAT A; template RAFL09-06-022) and β-CARBONIC ANHYDRASE 1 (ßCA1: fwd AAA AGG ATC CGC TCT TCA GAC AGG TAC TTC, rev TTT TCT CGA GCT ACA GCT TCC AAT GTA GTA TGG). These plasmids as well as those for CP12-1 and FERREDOXIN-DEPENDENT GLUTAMATE SYNTHASE 1 (Fd-GOGAT) were kindly provided by Kamel Chibani, Miriam Giesguth, Francesca Sparla (University of Bologna, Italy) and Wilena Telman. Expression was done in BL21 *E. coli* cells [12]. Recombinant proteins of 2-CysPRX WT and variants were produced using pET28a plasmids [14], except for LOX2, which was cloned in pET15b.

### 2.5. Isothermal Titration Microcalorimetry (ITC)

Recombinant proteins were reduced (10 mM DTT) or oxidized (1 mM H_2_O_2_) for 2 h and subsequently dialyzed overnight in buffer containing 35 mM HEPES, pH 8.0 with two buffer changes. The buffer from the last dialysis was taken as solution for the ITC titrations. Protein solutions were filtered through 0.45 µm membrane units and quantified using Bradford test after final dialysis. Thawed samples were degassed for 5 min. Isothermal titration microcalorimetry (ITC, MicroCal) with 1.4 mL cuvette volume was used for all experiments with reported settings [18]. Proteins of interest were injected into 2-CysPRX in order to avoid 2-CysPRX dissociation [18]. Concentrations and injection volumes were adjusted depending on the results from trial runs to optimize the experiment.

### 2.6. Far Western Overlay

Recombinant proteins were spotted in dilution series on nitrocellulose membranes (0.45 µm) and blocked with 1% (*w*/*v*) defatted milk powder in 50 mL Tris-buffered saline (TBS), pH 8. Spotted bait solutions were 5 µL of 0.5, 1.25, 2.5, 5, and 10 µM protein concentration. Spotted 2-CysPRXA served as positive and BSA as negative control. Association was achieved in 50 mM 3-(N-morpholino)propanesulfonic acid (MOPS), pH 8, 20% (*v*/*v*) glycerol, 10 mM MgCl_2_, either 2 mM DTT for reducing or 2 mM H_2_O_2_ for oxidizing conditions, and 20 µg 2-CysPRXA/mL (either WT, C54D, or C54S), overnight. The membranes were washed thrice with tris-buffered saline (TBS) plus 0.05% (*v/v*) Tween 20 (TBST), and incubated with antiserum directed against recombinant 2-CysPRX [9] at 1:5000 dilution for 3 h. Following three times washing with TBST, the secondary goat-anti rabbit antibody (1:50,000, Agrisera) conjugated to horseradish peroxidase was applied for 1 h. After three times washing with TBST, luminol turnover on the membrane was visualized by exposure of X-ray films. Negatives images were scanned with a Canon laser scanner and the greyness was quantified using the software ImageJ.

### 2.7. LIPOXYGENASE and ß-CARBONIC ANHYDRASE Activity Assays

Fatty acid oxidation was observed spectrophotometrically at 234 nm. Preincubation of recombinant and untreated LOX2 was done in 35 mM HEPES buffer at pH 8.0 for 15 min. This included 0.5 µM of LOX2 and 0.5 µM either reduced or oxidized 2-CysPRXA. The enzymatic reactions were initiated by adding 20 µM α-linolenic acid. The β-CARBONIC ANHYDRASE 1 activity assay was used according to [19,20]. Recombinant hisx6-tagged βCA1 was pretreated with 500 µM DTT for 30 min at room temperature for activation. The ßCA1 protein (50 nM) was added to 2 mL 20 mM Tris-HCl (pH 8.5 at 5°C) with 15 µg/mL phenolred (T127.2, Carl Roth, Karlsruhe, D) and 250 nM of the different 2-CysPRX variants (WT, C54D and C54S) with a final concentration of 50 nM. Then, 1.33 mL ice cold CO_2_-saturated water, including 15 µg/mL phenolred, was added to the cuvette to start the reaction at 2 °C. The reaction was monitored spectrophotometrically at 558 nm as decrease of absorbance of phenolred (Cary 3500 UV-Vis, Agilent). The Wilbur–Anderson Unit [19,21] was determined as the time span required for the solution pH to drop from 8.3 to 6.3.

## 3. Results

**Figure 1:**
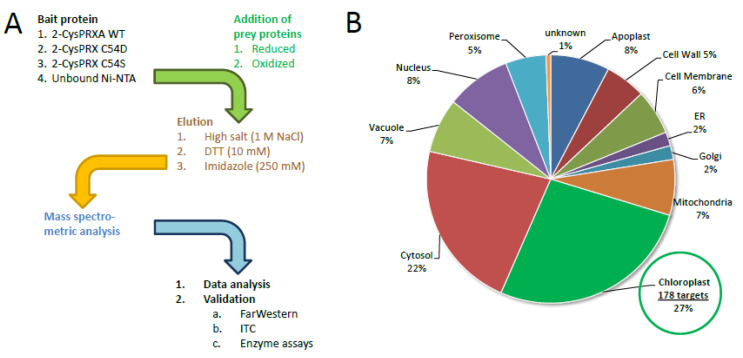
Workflow of the interactome study with 2-cysteine peroxiredoxin (2-CysPRX) variants and assignment of identified polypeptides to subcellular compartments. (**A**) Three variants of recombinant his6-tagged 2-CysPRXA (wild type (WT), C54S, C54D) were bound to Ni-nitrilotriacetic acid (NTA) sepharose. Unbound Ni-NTA-sepharose served as control. After binding of reduced or oxidized leaf proteins, washing, and step-wise elution, the peptides were identified by mass spectroscopy with technical replication. Data analysis employed UniRef90, MASCP Gator, and UniProt data bases. Some interaction partners were selected for further validation. (**B**) From 468 uniquely identified proteins compiled from all analyses, 178 or 27% were assigned to plastids. ER, endoplasmic reticulum; ITC, isothermal titration microcalorimetry; DTT, dithiothreitol.

This study aimed to identify interaction partners of 2-CysPRX in a redox state- and conformations-specific manner. Recombinant WT, C54S (mimicking the reduced form even in the presence of oxidants), and C54D protein (mimicking the hyperoxidized state) [14] was bound to Ni-nitrilotriacetic acid (Ni-NTA)-functionalized sepharose. Unbound Ni-NTA-sepharose served as control. Figure 1A summarizes the workflow with three different elution steps, namely, (1) with high salt for eluting electrostatically bound partners, (2) 10 mM dithiothreitol (DTT) for eluting polypeptides eventually bound by covalent disulfide bonding, and (3) imidazole to elute all residual material from the matrix. Polypeptides identified in the different fractions were included in the subsequent analysis if they were detected in at least two out of three independent pull downs and in both technical replicates, meaning in four out of six MS runs with a minimal Mascot Score of 25. Importantly, leaf proteins were applied either in the reduced or oxidized state. Therefore, the complexity of the experiment considered three variant, three elution regimes, and two redox-adjusted (in total 18) conditions measured from three independent experiments with two replicate determinations.

### 3.1. Identified Chloroplast Polypeptides

**Figure 2:**
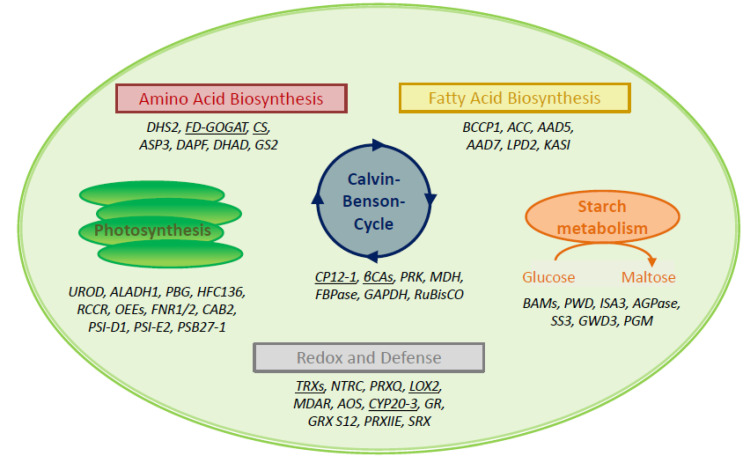
Simplified assignment of 49 identified polypeptides to six main chloroplast processes. Seven proteins each were assigned to the Calvin–Benson cycle (CBC) and associated processes, to starch metabolism and amino acid biosynthesis. Six polypeptides belonged to fatty acid synthesis. Eleven proteins each function in light reactions of photosynthesis and the combined group of redox regulation and stress defense. Underlined proteins are selected enzymes for validation. Abbreviations are as follows, starting with fatty acid biosynthesis clockwise: BCCP: BIOTIN CARBOXYL CARRIER PROTEIN OF ACETYL-COA CARBOXYLASE 1, ACC: BIOTIN CARBOXYLASE, AAD5: STEAROYL-[ACYL-CARRIER-PROTEIN] 9-DESATURASE 5, AAD7: STEAROYL-[ACYL-CARRIER-PROTEIN] 9-DESATURASE 7, LPD2: DIHYDROLIPOYL DEHYDROGENASE 2, KASI: 3-KETOACYL-ACYL CARRIER PROTEIN SYNTHASE I; BAM: β-AMYLASE, PWD: PHOSPHOGLUCAN WATER DIKINASE, ISA3: ISOAMYLASE 3, AGPase: GLUCOSE-1-PHOSPHATE ADENYLYLTRANSFERASE, SS3: STARCH SYNTHASE 3, GWD3: GLUCAN WATER DIKINASE 3, PGM: PHOSPHOGLUCOMUTASE; TRX: THIOREDOXIN, NTRC: NADPH-DEPENDENT THIOREDOXIN REDUCTASE C, PRXQ: PEROXIREDOXIN Q, LOX2: LIPOXYGENASE 2, MDAR: MONODEHYDROASCORBATE REDUCTASE, AOS: ALLENE OXIDE SYNTHASE, CYP20-3: CYCLOPHILIN 20-3, GR: GLUTATHIONE REDUCTASE, GRX S12: GLUTAREDOXIN S12, PRXIIE: PEROXIREDOXIN IIE, SRX: SULFIREDOXIN; UROD: UROPORPHYRINOGEN DECARBOXYLASE, ALADH1: δ-AMINOLEVULINIC ACID DEHYDRATASE 1, PBG: PORPHOBILINOGEN DEAMINASE, HCF136: HIGH CHLOROPHYLL FLUORESCENCE 136, RCCR: RED CHLOROPHYLL CATABOLITE REDUCTASE, OEE: OXYGEN-EVOLVING ENHANCER PROTEIN, FNR: FERREDOXIN-DEPENDENT NADPH REDUCTASE, CAB2: CHLOROPHYLL A-B BINDING PROTEIN, PSI-D1: PHOTOSYSTEM I REACTION CENTER SUBUNIT D1, PSII-E2: PHOTOSYSTEM II REACTION CENTER SUBUNIT E2, PSB27-1: PHOTOSYSTEM II REPAIR PROTEIN PSB27-H1; DHS2: PHOSPHO-2-DEHYDRO-3-DEOXYHEPTONATE ALDOLASE 2, FD-GOGAT: FERREDOXIN-DEPENDENT GLUTAMATE SYNTHASE, CS: CHORISMATE SYNTHASE, ASP3: ASPARTATE AMINOTRANSFERASE 3, DAPF: DIAMINOOPIMELATE EPMERASe, GS2: GLUTAMINE SYNTHETASE; CP12-1: CHLOROPLAST PROTEIN 12-1, βCA: β-CARBONIC ANHYDRASE, PRK: PHOSPHORIBULOKINASE, MDH: MALATE DEHYDROGENASE, FBPase: FRUCTOSE-1,6-BISPHOSPHATASE, GAPDH: GLYCERALDEHYD-3-PHOSPHATE DEHYDROGENASE, RuBisCO: RIBULOSE-1,5-BISPHOSPHATE CARBOXYLASE/OXYGENASE.

The interactome study was conducted with total leaf proteins. The majority of identified peptides (~73%) belonged to extrachloroplast compartments. This share compares to 58% of extrachloroplast proteins found in the previous study [16]. A set of 27% of all polypeptides localized to the chloroplast. Therefore, we focused on these 178 polypeptides as potential targets of 2-CysPRXA. They were compiled from the results with all variants, redox, and elution conditions (Figure 1B). The hits were cross-checked with the databases MASCP Gator and UniProt to minimize inaccuracies and false positives. The polypeptides were functionally assigned and grouped in metabolic pathways of the chloroplast 2-CysPRX (Figure 2). One group of proteins related to the function and biogenesis of photosynthetic light reaction and included photosystem I subunits and repair proteins (PSI-D1/-E1, PSB27-1), redox reactive proteins (HSF136, FD-dependent NADP REDUCTASE (FNR1/2)), and enzymes involved in chlorophyll synthesis (UROPORPHORINOGEN DECARBOXYLASE (UROD)). Proteins with function in the CBC and associated activities comprised FRUCTOSE-1,6-BISPHOSPHATASE (FBPase), the intrinsically disordered regulator CP12-1, PHOSPHORIBULOKINASE (PRK), β-CARBONIC ANHYDRASE1 (ßCA1), and GLYCERALDEHYDE-3-PHOSPHATE DEHYDROGENASE (GAPDH). A small set concerned starch metabolism and included STARCH SYNTHASE 3 (SS3), ADP GLUCOSE PYROPHOSPHORYLASE (AGPase), ISOAMYLASE 3 (ISA3), and β-AMYLASE 2/3 (BAM2/3). Proteins of amino acid metabolism were FERREDOXIN-dependent GLUTAMATE SYNTHASE 1 (FD-GOGAT) and CHORISMATE SYNTHASE (CS). ACETYL-CoA CARBOXYLASE CARBOXYL TRANSFERASE (ACCase) and STEAROYL-ACP DESATURASE 7 associate with fatty acid biosynthesis. Redox and hormone-related proteins comprised PRX Q; PRXIIE; several TRXs; and two enzymes of jasmonic acid synthesis, namely LIPOXYGENASE 2 (LOX2) and ALLENE OXIDE SYNTHASE (AOS). Further analysis using functional annotations of tair11 showed a high share of interaction partners with function in photosynthesis (31 proteins); protein homeostasis (28 proteins); and redox signaling, lipid, and amino acid metabolism (11, 12, and 11 proteins, respectively). A set of 26 proteins represented carbon metabolism (Table S1).

**Figure 3:**
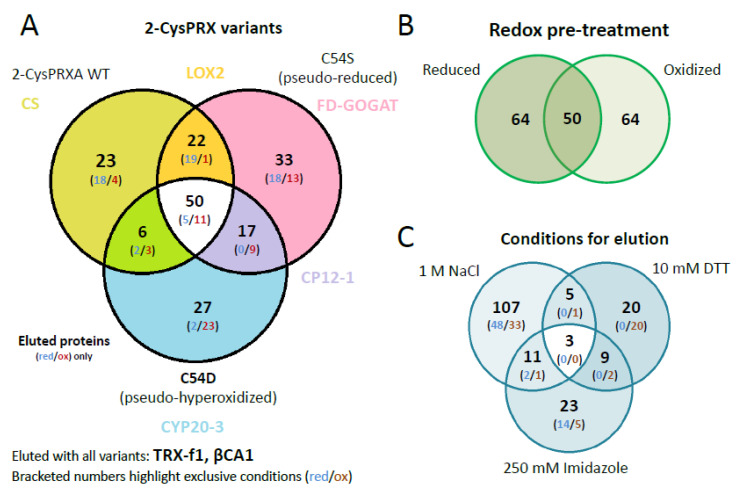
Venn diagrams depicting overlapping and unique 2-CysPRX interactors. (**A**) Comparison of the entire group of interactors of each variant. The set binding to all variants comprised 50 proteins of 178. The group of unique binders to WT, C54S, and C54D consisted of 23, 33, and 27 polypeptides, respectively. (**B**) The compiled groups of redox-specific interactions comprised 64 polypeptides binding under reducing and oxidizing conditions each, and 50 polypeptides binding under both conditions. (**C**) Comparison of the proteins eluted with high salt (NaCl), under reducing conditions (DTT) and as complex with 2-CysPRX by washing with imidazole. The largest group of unique polypeptides eluted with 1 M NaCl and included 107 polypeptides.

The identified polypeptides were categorized according to unique and overlapping interactions with the different variants (Figure 3A). A set of 23 polypeptides specifically bound to WT, 35 to C54S, and 27 to C54D. A group of 50 polypeptides bound to all variants (Table S2). Analyzing the unique interactors revealed that redox-active targets of 2-CysPRX like the NADPH-dependent THIOREDOXIN REDUCTASE C (NTRC) and sulfiredoxin (SRX), mainly responsible for regeneration of 2-CysPRX, were only found in WT elutions with both catalytic cysteines available.

Interestingly, C54D bound a large share of proteins involved in photosynthesis and redox regulation such as reduced glutathione (GSH) homeostasis. Exclusive interactions concerned polypeptides associated with thylakoids including PRXQ, HCF136, PLASTOCYANIN (PC), or RED CHLOROPHYLL CATABOLITE REDUCTASE (RCCR). Moreover, proteins of glutathione synthesis and homeostasis and jasmonic acid synthesis bound to C54D, for example, ALLENE OXIDE SYNTHASE (AOS), CYCLOPHILIN 20-3 (CYP20-3), and GLUTATHIONE REDUCTASE (GR). These proteins exclusively eluted from C54D with NaCl after adjustment to the oxidized state.

Proteins involved in other metabolic pathways selectively interacted with single variants, like enzymes of fatty acid, carbon, and starch metabolism or translation. Different enzymes of lysine biosynthesis represented unique partners of each 2-CysPRX variant, which eluted at high NaCl concentrations. Enzymes of chorismate, arginine, or glutamate synthesis associated with C54S or WT 2-CysPRX and eluted after addition of DTT. Unique variant interactions may provide insight into conformation-specific functions of 2-CysPRX.

Our unique experimental design with mimic variants opened the opportunity to incubate the pseudoreduced conformation of C54S with oxidized or reduced protein extracts. This would not be possible with wildtype 2-CysPRX because reducing or oxidizing conditions would affect the redox state of both the bait 2-CysPRX and the leaf proteins as prey. The set of 178 polypeptides revealed a similar distribution with 64 targets identified under oxidizing and reducing conditions, respectively, and 50 polypeptides as overlap of both sets (Figure 3B).

Successive elution steps separated electrostatically bound interactors (1 M NaCl), redox conformation- or mixed disulfide-related binding partners (10 mM DTT), and strong binders by eluting the Ni-NTA-bound 2-CysPRX together with the associated polypeptides (250 mM imidazole) (Figure 3C). High salt washing released 107 proteins, DTT 20 proteins, and imidazole 24 proteins. Only two targets, an ADENYLOSUCCINATE LYASE and GLYCERALDEHYDE 3-PHOSPHATE DEHYDROGENASE A SUBUNIT 2 (GAPA2), were present in all eluted fractions. Elution by thiol reduction revealed many target proteins with function in photosynthesis or its regulation, namely OXYGEN-EVOLVING ENHANCER PROTEIN 1-1, PRXQ, NTRC, GAPDH, and PORPHOBILINOGEN DEAMINASE as part of tetrapyrrole synthesis. Proteins released from the column in the final step of eluting with imidazole function in photosynthesis like PRK, RuBisCO, ßCA2, and TRANSKETOLASE 1, but also in starch metabolism, which includes β-AMYLASE (BAM2/3), ISOAMYLASE 3 (ISA3), GLUCOSE-1-PHOSPHATE ADENYLYLTRANSFERASE, and PHOSPHOGLUCAN WATER DIKINASE (PWD).

A set of 78 proteins exclusively eluted in only one redox- or variant-specific condition (Table 1). Here, some peculiar trends were seen in specific binding of polypeptides to 2-CysPRX under either reducing or oxidizing conditions. The number of unique proteins in the NaCl fraction under reducing condition decreased in the order of WT over C54S to C54D (17 vs. 11 vs. 1) and, simultaneously, more proteins bound under oxidizing conditions (2 vs. 6 vs. 17). The set size of weakly bound proteins (NaCl elutions) exceeded that released by redox shifts (DTT elutions) or strongly bound proteins (imidazole elutions). Only about one-third of all targets were uniquely identified in the functional groups of photosynthesis and protein homeostasis and two-thirds were associated with 2-CysPRX under more than one condition. This contrasts the functional groups amino acid biosynthesis, fatty acid biosynthesis, C-metabolism, or redox with around 60% of unique interactions. Nevertheless, all these functional groups were present in both categories of uniquely and commonly bound proteins. None of the variants, elution conditions, or redox states exclusively selected all components of a specific pathways identified as 2-CysPRX interaction partners.

### 3.2. Validation of Potential Targets

The use of site-directed mutated variants with stabilized conformation offered a novel opportunity for studying the 2-CysPRX interactome, for example, using a pseudoreduced variant of 2-CysPRX together with oxidized prey proteins. The next step aimed to validate some of the interactions using Far Western, ITC, and enzyme assays for LOX2 and ßCA1. Previous transcriptome and metabolite data sets for *2cysprxA/B* [12,22,23] were taken into account to select interesting candidates for validation. The regulator proteins CP12-1 and Trx-f1, as well as ßCA1, represented carbohydrate metabolism. FD-GOGAT and CS were examples from amino acid biosynthesis. LOX2 and LOX4 catalyze the committed step of oxylipin synthesis and Cyp20-3 is an effector protein in oxylipin signaling. The eight proteins were produced as recombinant *hisx6*-tagged proteins in *E. coli*.

**Figure 4:**
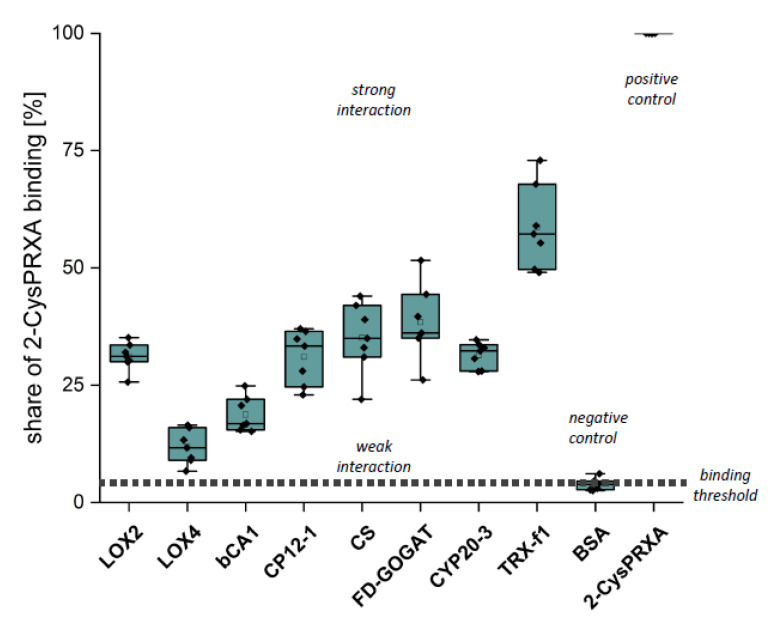
Binding of 2-CysPRX to recombinant polypeptides analyzed by Far Western blotting. Dilution series of recombinant proteins of β-CARBONICANHYDRASE (ßCA1), CHORISMATE SYNTHASE (CS), CHLOROPLAST REGULATOR (CP12-1), CYCLOPHILIN 20-3 (CYP20-3), FEREDOXIN-dependent GLUTAMINE-OXOGLUTARATE AMINOTRANSFERASE (FD-GOGAT), 13-LIPOXYGENASE (LOX2 and LOX4), and THIOREDOXIN-f1 (TRXf1). BOVINE SERUM ALBUMIN (BSA) served as negative and 2-CYSTEINE PEROXIREDOXIN (2-CysPRX) as positive control. The nitrocellulose membrane with the spotted proteins was incubated with 20 µg/mL 2-CysPRX in association buffer overnight. Box plots display the result from six independent Far Western blots. The data are presented as box plots showing all individual results, the median, the first and third quartiles, and the data range.

The first verification step consisted of Far Western overlays. Recombinant proteins were spotted on a nitrocellulose membrane, incubated with 2-CysPRX variants and then with anti-2-CysPRX antiserum and secondary antibody, and subjected to luminescent detection. In our hands, this application was not sensitive enough to discriminate interactions of 2-CysPRX variants. Thus, the presented data (Figure 4) sum up all binding intensities and represent estimates of interaction strength (Figure 4). Spotted 2-CysPRXA WT served as positive control and BSA as negative control. CYP20-3 is a proven interactor of 2-CysPRX [18]. TRX-f1 achieved the highest signal intensity with about 60% of the 2-CysPRX control. The signal intensity with BSA near 4% was set as the threshold for non-specific interaction. In each case, the intensity increased with the amount of spotted proteins. Fd-GOGAT, CS, CP12-1, Cyp20-3, and LOX2 displayed a similar signal of about 30%. The signal for ßCA1 ranged around 17% and LOX4 near 11%.

**Figure 5:**
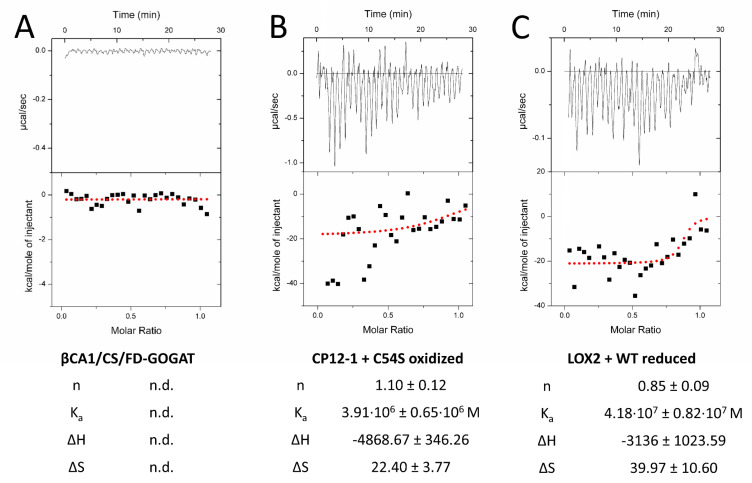
Quantification of the interaction of 2-CysPRX with five interactors by isothermal titration calorimetry (ITC). Recombinant proteins were redox-adjusted and dialyzed for optimal performance. The dialyzed polypeptides were titrated into 2-CysPRXA to circumvent dissociation effects of 2-CysPRX. ßCA1, CS, and FD-GOGAT were not responsive in any variant combination (**A**), while CP12-1 (**B**) and LOX2 (**C**) gave parameters fitting to a weak interaction. Displayed ITC graphs represent one reading, while given values are means of a minimum of four independent measurements. Abbreviations: n: molar ratio; n.d.: not determined; K_a_: association constant; ΔH: reaction enthalpy; ΔS: change in entropy.

We next tested the interaction by titration microcalorimetry (ITC) (Figure 5). ITC uncovers interactions in solution and provides quantitative thermodynamic data like binding affinity and stoichiometry. Recombinant prey proteins were titrated into the 2-CysPRX-containing cuvette. Injection of CS, FD-GOGAT, and ßCA1 into either 2-CysPRXA WT or C54S gave signals near background (Figure 5A). Injection of CP12-1 achieved positive read-out with C54S under oxidizing conditions (Figure 5B), and LOX2 with 2-CysPRX WT under reducing conditions. Calculated stoichiometry was close to one in both cases. Enthalpy and entropy values combined to an exergonic reaction (ΔG < 0) for CP12-1 and LOX2, proving the spontaneous interaction.

**Figure 6:**
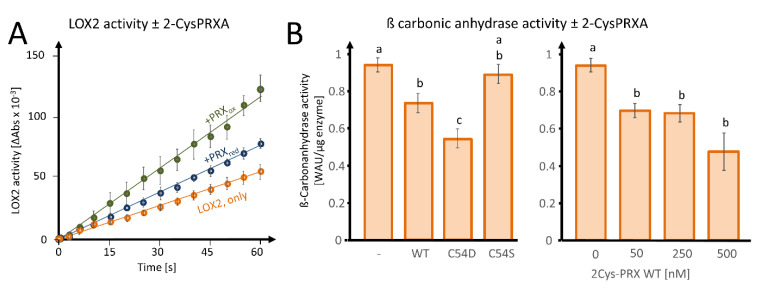
Effects of 2-CysPRX on LIPOXYGENASE 2 (LOX2) and ß-CARBONIC ANHYDRASE activities. (**A**) Effect of reduced or oxidized 2-CysPRX (0.5 µM monomer concentration) on LOX2 activity (0.5 µM recombinant protein). Reduced 2-CysPRXA improves α-linolenic acid oxidation activity slightly compared with non-treated LOX2 alone (green). Oxidized 2-CysPRXA doubled the oxidation rate of α-linolenic acid. (**B**) Effect of WT, C54S, and C54D protein of 2-CysPRX (250 nM) on ßCA1 (50 nM) activity monitored spectrophotometrically using the pH indicator phenolred. Experiments were conducted with two independently generated recombinant protein lots and represent means ± SE of *n* ≥ 10 (variants) and *n* ≥ 8 (concentration-dependency). Data were analyzed for statistical significance of difference using one way analysis of variance (ANOVA) and post-hoc Tukey HSD (honestly significant difference).

In order to investigate the effect of 2-CysPRX binding on the biochemical activity, we tested LOX2-catalyzed peroxidation of α-linolenic acid (αLeA) in the presence of either reduced or oxidized 2-CysPRX (Figure 6A). The preform of nuclear encoded LOX2 contains a transit peptide of 79 amino acids for plastid targeting being cleaved upon import. The mature form of LOX2 has an N-terminal PLAT-domain of 120 amino acids followed by the lipoxygenase domain of 697 amino acids. Figure S1 depicts the ribbon structure from two opposed view angles. LOX2 has 10 Cys residues, 3 of which are predicted to be conserved during evolution with a Cys score of 0.71 (Cys181, Cys561) and 0.64 (Cys274) and *p*-values of 0.04 and 0.12 (Figure S1B; online tool ConCysFind: https://bibiserv.cebitec.uni-bielefeld.de/concysfind). Supplementation of the lipoxygenase assay with 2-CysPRX increased the rate of αLeA peroxidation 1.4-fold in the case of the reduced form and 2.4-fold in the case of the oxidized form.

As a second enzyme, we tested the effect of fivefold excess WT, C54S, and C54D forms of 2-CysPRX on activity of 50 nM recombinant ßCA1 and observed an inhibition of the acidification rate owing to decreased formation of carbonic acid from CO_2_ (Figure 6B). The pseudo-hyperoxidized form C54D inhibited the activity more than the WT form, while the pseudoreduced form C54S was ineffective. Inhibition increased with the 2-CysPRX WT amount. It should be noted that only the reduced form of ßCA1 was active; therefore, these tests could only be performed under reducing conditions, as outlined in Section 2.

## 4. Discussion

### 4.1. 2-CysPRX Protein–Protein Interactions: Comparing Current Knowledge and New Results

A deep understanding of many biological processes depends on knowledge of molecular interactions. To this end, methods like yeast-two-hybrid screens, bifunctional fluorescence complementation, and high-throughput mass spectrometry have pushed this research field. Previous studies have revealed several interaction partners of 2-CysPRX, initially mostly with a focus on biochemical re-reduction of the oxidized form, in particular TRX-m [24], TRX-x [25], NTRC [26], and CHLOROPLASTIC DROUGHT-INDUCED STRESS PROTEIN of 32 kDa (CDSP32) [27]. These interactions were considered to be of catalytic and short-lived nature. Caporaletti et al. [28] described the non-reductive activation of chloroplast FBPase by 2-CysPRX. Muthuramalingam et al. [17] detected 2-CysPRX in photosystem II complexes and CYP20-3 acted as interactor of 2-CysPRX [18]. These interactions were of longer-lasting duration and high stability. The results from our study overlap with this list with NTRC; CYP20-3; photosystem II-related complexes; and various TRXs like m1, m4, and f1 bound to 2-CysPRX immobilized on column materials. 

Cerveau et al. [16] conducted an interactome study based on immunoprecipitation of 2-CysPRX with attached interactors from *A. thaliana* wildtype leaf extracts, and as control from *2-cysprxAB* knockout plants. The immuno-pull-out included a step of 30 min shaking of the mix of antibody-resin and leaf extract. The work did not address redox- and conformation-dependent interactions. This well-conceived paper elucidated the vast interaction potential of 2-CysPrx. From the set of 158 identified proteins, 67 had an annotated localization in plastids. The relative share of 42% of plastid proteins among all identified polypeptides exceeded our result of 27% of plastidic proteins among all polypeptides. 

The comparison of the list from Cerveau et al. [16] with our hit list revealed an overlap of only 17 proteins. The overlapping group consists of representatives from photosynthesis (FBPase, RuBisCO, FNR), amino acid synthesis (Fd-GOGAT, PHOSPHO-2-DEHYDRO-3-DEOXYHEPTONATE ALDOLASE 2), and redox regulation (PRXQ, GR, GSH-S-TRANSFERASE F8) (Table 1). 

Several global approaches used proteins such TRXs and glutaredoxins (GRX) as bait and eluted 2-CysPRX as prey, for example, by affinity trapping with a Cys_R_–Ser mutated TRX variant [29] and the poplar GRX S12 [30]. Schürmann and Buchanan [31] summarized these results for binding and interaction partners of TRX. 2-CysPRX was found as a TRX target from spinach, pea, sorghum, maize, wheat, and potato. TRXs regulate committed steps of the CBC, fatty acid biosynthesis, starch metabolism, energy metabolism, oxidative pentose phosphate cycle, protein folding, and redox regulation. Thus, there is a major match with targets described here like UROD, BAM, α-GLUCAN WATER DIKINASE (GWD), FBPase, GAPDH subunit B, CP12, PRK, MDH, ATP-DEPENDENT CLP PROTEASE, CA, and ACETYL-CoA CARBOXYLASE subunit A. The match between TRX targets and 2-CysPRX interactors may indicate that leaf TRXs contained in the extracts mediated some of the interactions between 2-CysPRX and target proteins found in our study. Such trilateral interactions should be testable in future experiments.

### 4.2. Lack of 2-CysPRX in Plants: A Focus on Transcript and Metabolite Level 

Arabidopsis plants lacking both isoforms of 2-CysPRX A and B reveal decreased growth, altered transcriptomes, metabolite levels, and redox state. Awad et al. [22] proposed higher ROS accumulation and lower photosynthetic CO_2_ fixation as a cause of retarded growth of *2-cysprxAB* plants. Later, Vaseghi et al. [12] reported a peroxide-dependent TRX-oxidase function for 2-CysPRX needed for adjusting the enzyme activities in the CBC and malate valve. In this mechanism, TRX functions as redox transmitter for oxidation and inactivation according to the light requirements. In line with this function, it is shown here that TRX-f1/-m1/-m4, several CBC enzymes, and MDH bind to 2-CysPRX.

Amino acid and carbohydrate levels are altered in *2-cysprxAB* compared with WT in moderate and high light [23]. Several amino acids accumulate in *2cysprxA/B* in growth light compared with WT. In particular, Phe reaches thrice the amount of WT, but Gly and Ala amounts are also elevated. Upon transfer to high light, some amino acid levels increase like Gly, Leu, Gln, and Phe in WT, but this increase was insignificant for Phe or lower for Gly, Leu, and Gln in *2-cysprxAB*. Phe is a product of the shikimate pathway with chorismate as an intermediate [22]. Enzymes of this pathway interacted with 2-CysPRX, that is, CHORISMATE SYNTHASE and PHOSPHO-2-DEHYDRO-3-DEOXYHEPTONATE ALDOLASE 2.

*2-cysprxAB* grow slower than WT and accumulate less glucose and fructose [22]. Upon 6 h exposure to high light, *2-cysprxAB* also have lower sucrose and maltose amounts than WT. It is tempting to hypothesize that these changes in carbohydrate dynamics are linked to the interaction reported here with FBPase, PRK, GAPDH, and CP12-1, as well as PHOSPHOGLUCAN PHOSPHATASE, PWD, GWD1, ß-AMYLASE 2/3, ISA3, SS3, and PHOSPHOGLUCOMUTASE.

Several α- and β-AMYLASES are redox-regulated in most cases by TRX [32,33]. Other enzymes of starch metabolism, in both the direction of synthesis and degradation, also display redox sensitivity [34]. Starch synthesis as light energy-dependent pathway relies on proper and fast regulation to manage energy supply and storage. Enzymes like SS3 and GWD1 are prone to redox transitions, but evidence for this type of regulation is missing for PWD. Starch-degrading proteins like STARCH EXCESS, α- and β-AMYLASEs, as well as ISOAMYLASEs are redox-regulated and were found as interactors of 2-CysPRX.

Interestingly, most of the starch-synthesizing enzymes (SS3, GWD1) interacting with 2-CysPRX associated with the chaperone-like C54D variant, while most catabolic enzymes eluted from C54S (BAM3, ISA3). The intriguing interaction of 2-CysPRX with enzymes of starch metabolism may suggest a new level of regulation in carbohydrate metabolism. In addition to tuning of CBC by 2-Cys PRX as TRX oxidase [12,13], 2-CysPRX may be involved in modulating synthesis and degradation of transitory starch. Starch synthesis in the daily light phase usually accelerates during the late morning hours when the initially used export pathways tend to saturate. This may coincide with increased ROS formation. Starch degradation occurs at night when ROS production is low. Interestingly, all starch ana- and katabolic enzymes analyzed for redox sensitivity are reductively activated, which seems counterintuitive as one would hypothesize for antagonistic regulation of synthesis and degradation [34]. To understand the significance of the redox regulatory network and the function of 2-CysPRX in starch metabolism, it would be interesting to assess the diurnal changes in stromal redox state, ROS production, 2-CysPRX conformation, and activation state of involved enzymes. It will have to be explored whether the association between starch metabolizing enzymes and 2-CysPRX plays an activating, inhibitory, or stabilizing role.

### 4.3. Novel Interaction Partners of 2-CysPRX and Redox Regulation

‘omics’-approaches assist in identifying previously undiscovered mechanisms and dependencies. Emerging lists of interaction partners require validation. Therefore, we selected ßCA1, CP12-1, and TRX-f1 as examples from carbon metabolism; FD-GOGAT and CS from amino acid metabolism; and LOX2, LOX4, and the regulator CYP20-3 from oxylipin synthesis. Carbonic anhydrases improve carbon dioxide availability for RIBULOSE-1,5-BISPHOSPHATE CARBOXYLASE, CP12-1 is a redox-dependent regulator complexing and inactivating GAPDH and PRK, and TRX-f1 controls many CBC enzymes. The reduced glycine level and higher Ser/Gly ratio in *2cysprxA/B* point to a role of 2-CysPRX in adjusting photorespiration [23]. β-type CAs are the predominant carboanhydrases in plants [35]. Kikutani et al. [36] provided first evidence for TRX-linked redox regulation of ßCA1 in the diatom *Phaeodactylum tricornutum*. The Far Western blot confirmed binding of ßCA1 to 2-CysPRX; however, ITC did not reveal detectable heat release when ßCA1 was injected into 2-CysPRX solution. The lack of ITC signal may be owing to balanced exothermic and endothermic reactions during binding or missing mediators such as TRX. Supplementation of the ßCA1 activity test with WT or pseudohyperoxidized C54D variant of 2-CysPRX inhibited the ßCA1-dependent hydration reaction of CO_2_ (Figure 6). ßCA1 participate in CO_2_-dependent stomata regulation, which should be investigated in 2-cysprxAB mutants [37].

CP12-1 is a small and unstructured regulatory protein. Its association with the inactive PRK-GAPDH-CP12 complex depends on the redox state of CP12. TRX-dependent reduction dissociates the inhibitory complex in the light [38,39,40,41]. CP12-1 bound to the C54D and C54S variants during incubation with oxidized leaf proteins. Far Western and ITC confirmed its interaction with 2-CysPRX. Interestingly, the reported complex partners PRK and GAPDH bound and eluted with similar specificity as CP12-1. PRK and GAPDH formed strong complexes with 2-CysPRX, as they only eluted with imidazole. GAPDH bound to all variants of 2-CysPRX upon incubation with oxidized leaf extract and eluted with DTT. 2-CysPRX may be part of a complex with tightly bound PRK and GAPDH. The latter interaction is redox-dependent on its own, but does not depend on a particular 2-CysPRX conformation. In addition oxidized CP12 binds to 2-CysPRX. The working hypothesis for future work may propose a dual role for 2-CysPRX; the decamer binds to the GAPDH-PRK-CP12 complex as chaperone or 2-CysPRX participates as TRX oxidase in redox regulation during light–dark transition, reverting the TRX-dependent activation [42].

Several amino acids like Gly, Glu, Ala, Tyr, and Phe are elevated in *2-cysprxAB* under normal growth conditions [23]. Upon transfer to high light, Phe, Tyr, and Gln levels increase in WT, but *2-cysprxAB* fails to respond in a similar manner. Glu amounts decrease in *2-cysprxAB* in response to increased light intensity. Phe and Tyr are synthesized in the shikimate pathway, and Glu and Gln participate in NH_4_^+^-reassimilation during photorespiration (Glu and Gln). Both processes depend on specific chloroplast enzymes, namely CS catalyzing the last step in shikimate synthesis and FD-GOGAT as well as GLUTAMINE SYNTHETASE (GS) in Glu and Gln synthesis, respectively. All three enzymes associated with 2-CysPRX under distinct conditions: GS bound to C54S independent on redox conditions and eluted with imidazole indicating strong redox-independent interaction. The observation that reduced FD-GOGAT binds to C54S and elutes with imidazole implies strong interaction under reducing conditions. The association between reduced CS and WT 2-CysPRX is relatively weak and partly depends on redox state, as revealed by the dissociation with NaCl and DTT. Analysis of Far Western confirmed the interaction between 2-CysPRX and both CS and FD-GOGAT, but could not be detected by ITC.

FD-GOGAT and GS are well-described targets of TRX-dependent regulation, similar to CBC enzymes, ADP GLUCOSE PYROPHOSPHORYLASE involved in starch synthesis, and ACETYL-CoA-CARBOXYLASE (ACCase) in fatty acid synthesis [29,43]. In a converse manner, reliable information on redox regulation of CS is unavailable. Analysis of CS protein structure and phylogenetic Cys conservation identified eight Cys in *Arabidopsis thaliana*, seven of which are highly conserved in the plant kingdom (AROC_ARATH, Figure S2, online tool ConCysFind: bibiserv.cebitec.uni-bielefeld.de/concysfind). The degree of Cys conservation may indicate thiol- and TRX-dependent regulation. Future work should address the possible redox regulation of CS and the functional significance of its interaction with 2-CysPRX.

Oxylipin signaling plays a major role in stress defense and redox regulation of plants [44]. 13-LOX catalyzes the first reaction of O_2_-dependent dioxygenation of α-linolenic acid in chloroplasts producing an alkylhydroperoxide, which is converted to oxophytodienoic acid (OPDA) by ALLENEOXIDE SYNTHASE (AOS) and ALLENEOXIDE CYCLASE (AOC). OPDA binds to CYP20-3, which then activates chloroplast CYSTEINE SYNTHASE complex [23,45,46]. 2-CysPRX in turn binds to CYP20-3 in a redox-dependent manner [18]. CYP20-3 was identified as interaction partner of 2-CysPRX in this study, but surprisingly as binding partner of the pseudohyperoxidized form C54D under oxidizing conditions. This combination was not studied before. In previous investigations, 2-CysPRX associated with Cyp20-3 under reducing conditions [18]. Both the reduced and hyperoxidized form adopt the decameric state.

LOX2 is the by far most abundant 13-LOX in the chloroplast with 2304 ppm compared with the other isoforms with <10 ppm in leaves [44]. In addition to LOX2, AOS, the second enzyme of OPDA synthesis, also bound to 2-CysPRX under oxidizing conditions (Figure 2, Table S3). Some first experimental evidence suggests the existence of a supramolecular complex of LOX, AOS, and AOC [47], and 2-CysPRX may play a role in the formation of this complex involved in substrate channeling. ConCysFind identified three phylogenetically conserved Cys in LOX2 (Figure S1). This is suggestive of redox regulation of LOX2. Elution of LOX2 from WT and C54S with NaCl indicates interaction with 2-CysPRXA driven by electrostatic bonds. Both reduced and oxidized 2-CysPRX activated LOX2 activity (Figure 6). OPDA, the end-product of the pathway, promotes Cys and glutathione synthesis and enables redox homeostasis [46]. The multiple mechanistic interferences between components of Cys synthesis, OPDA synthesis, Cyp20-3, OPDA, and 2-CysPRX may realize negative and/or positive feedback regulation and await experimental dissection.

The use of leaf proteins as prey enables an untargeted search for interactors. On the other hand, the complex composition of leaf proteomes fosters the formation of secondary interactions where a primary interactor of 2-CysPRX attracts other proteins. Their identification by MS then pretends interaction with 2-CysPRX. Although the discovery of such 2-CysPRX-targeted complexes was not a primary goal of this work, they are of significant interest as pointed out above for oxylipin synthesis and LOX2. The high overlap between our results and the list of reported TRX targets (Table 2) may suggest secondary complex formation between 2-CysPRX, TRXs, and TRX targets. TRX-f1/-m1/-m4 eluted with NaCl. Secondary interactors trapped by 2-CysPRX via TRX should also elute in the NaCl step like FBPase, MDH, and CP12-1. Therefore, it will be an important challenge to separate TRX-mediated interaction of 2-CysPRX and other secondary interactions from direct primary interactions.

## 5. Conclusions

Using redox-mimic variants of 2-CysPRX and redox-adjusted incubation of prey and bait, this report describes selective redox interactions of 2-CysPRX and proteins/enzymes of important metabolic pathways like CBC, amino acid and fatty biosynthesis, carbohydrate metabolism, and protein homeostasis. The tentative validation of interactions worked out well with Far Western, where all tested recombinant proteins revealed binding with 2-CysPRX stronger than with BSA. ITC validated the interaction with CP12-1 and LOX2. The effect of 2-CysPRX on LOX2 and ßCA1 activities further substantiated these particular interactions, the physiological significance of which was discussed above and should be addressed in future work.

The long list of interactors identified in this study substantiates the previously proposed multiple roles of 2-CysPRX as redox sensor, TRX oxidase, chaperone, and regulatory interactor [17]. Previously described changes in transcriptomes and metabolomes in *2-cysprxAB* plants [23] support the significance of some of the interactions described here. Several directions for future work arise from this work.

(a) There is a need to distinguish primary interactors of 2-CysPRX from secondary interactions and macromolecular complexes. 2-CysPRX may function as scaffold of complex formation as in the case of LOX2/AOS/AOC.

(b) The overlap between TRX targets and components of the 2-CysPRX interactome indicates formation of complexes with TRXs as mediators. Such mediator function can be scrutinized by reconstitution in vitro with recombinant proteins. It will also be important to explore the function of such complexes, for example, for efficient oxidation of TRX target proteins by H_2_O_2_ in the presence of 2-CysPRX [12,13].

(c) The use of site-directed mutated variants of 2-CysPRX with “frozen” conformation included C54S and C54D in this study. It should be expanded to understand the dynamics of redox-dependent transitions in 2-CysPRX conformation. The F84R variant is unable to decamerize [14]. Its analysis in a similar proteomics approach may allow us to distinguish the function as the oligomeric form as interactor and chaperon, and peroxidase as the F84R-variant displays a 3- to 4-fold higher thiol peroxidase activity.

(d) Functional assays need to be established to scrutinize interactions described in this report.

## Figures and Tables

**Table 1:** Overview of unique proteins eluted under all conditions. WT, wild type; DTT, dithiothreitol.

	2-CysPRXA WT		C54S (Pseudo-Reduced)		C54D (Pseudo-Hyperoxidized)	
	Reduced	Oxidized	Reduced	Oxidized	Reduced	Oxidized
**1 M NaCl**	30S ribosomal protein S17 (AT1G79850)	HTPA synthase 2 (AT2G45440)	30S ribosomal protein S6 alpha (AT1G64510)	MECDP synthase (AT1G63970)	Acyl-ACP thioesterase ATL3 (AT1G68260)	HTPA synthase 1 (AT3G60880)
	DAHP synthase 2 (AT4G33510)	Elongation factor 1 alpha (AT4G20360)	TRAF-like family protein (AT3G28220)	HTPA reductase 1 (AT2G44040)		Adenylosuccinate synthetase (AT3G57610)
	CDPME kinase (AT2G26930)		Ferredoxin--NADP reductase (AT1G20020)	50S ribosomal protein L27 (AT5G40950)		Allene oxide synthase (AT5G42650)
	50S ribosomal protein L29 (AT1G79850)		Protein MET1 (AT1G55480)	50S ribosomal protein L5 (AT4G01310)		AT5g64380/MSJ1_22 (AT5G64380)
	60S ribosomal protein L26-1 (AT3G49910)		Glutathione S-transferase F8 (AT2G47730)	ATPase alpha subunit (AtCg00120)		Dihydroxy-acid dehydratase (AT3G23940)
	PLAT domain-containing protein 1 (AT4G39730)		Photosystem I reaction center subunit II-1 (AT4G02770)	Monodehydroascorbate reductase 6 (AT1G63940)		E-Z type HEAT repeat- protein (AT3G62530)
	Chlorophyll a-b binding protein 2 (AT1G29920)		Protease Do-like 1 (AT3G27925)			Glutathione reductase (AT3G54660)
	Chorismate synthase (AT1G48850)		Protein COLD-REGULATED 15A (AT2G42540)			HAD-superfamily hydrolase, 5’-nucleotidase (AT5G48960)
	Delta-aminolevulinic acid dehydratase 1 (AT1G69740)		Protein COLD-REGULATED 15B (AT2G42530)			Peptidyl-prolyl isomerase CYP20-3 (AT3G62030)
	Malate dehydrogenase (AT3G47520)		Putative protein (At2G22230)			Photosystem II factor HCF136 (AT5G23120)
	Phosphoglucan phosphatase LSF1 (AT3G01510)		Soluble pyrophosphatase 6 (AT5G09650)			Plastidial pyruvate kinase 3 (AT1G32440)
	Probable protein phosphatase 2C 62 (AT4G33500)					Plastocyanin major isoform (AT1G20340)
	Pyruvate dehydrogenase E1 subunit beta-3 (AT2G34590)					Red chlorophyll catabolite reductase (AT4G37000)
	Single-stranded DNA-binding protein WHY1 (AT1G14410)					Ribose-P pyrophosphokinase 3 (AT1G10700)
	Transaldolase-like protein (AT1G12230)					Starch synthase 3 (AT1G11720)
	Uncharacterized protein (AT2G27680)					Stearoyl-[acyl-carrier-protein] 9-desaturase 5 (AT3G02630)
						Thiosulfate sulfurtransferase 16 (AT5G66040)
**10 mM DTT**		FGGY family of carbohydrate kinase (AT4G30310)		Formate--tetrahydrofolate ligase (AT1G50480)		Phosphofructokinase 5 (AT2G22480)
		NADPH-dep. thioredoxin reductase C (AT2G41680)		NADP-dependent malic enzyme 2 (AT5G11670)		Bifunctional protein FolD 2 (AT3G12290)
				Oxygen-evolving enhancer protein 1-1 (AT5G66570)		Peroxiredoxin Q (AT3G26060)
						Plastidial pyruvate kinase 2 (AT5G52920)
						Thylakoid lumenal 19 kDa protein (AT3G63540)
**250 mM Imidazole**	Sulfiredoxin (AT1G31170)		Arginase 2 (AT4G08870)	30S ribosomal protein S10 (AT3G13120)	Phosphoglucan, water dikinase (AT5G26570)	
	Transketolase-1 (AT3G60750)		BCCP of acetyl-CoA carboxylase 1 (AT5G16390)	Beta-amylase 3 (AT4G17090)		
			Biotin carboxylase (AT5G35360)	Isoamylase 3(AT4G09020)		
			Ferredoxin-dependent glutamate synthase 1 (AT5G04140)			
			GDSL esterase/lipase ESM1 (AT3G14210)			
			Myrosinase 2 (AT5G25980)			
			AIR carboxylase like protein (AT2G37690)			

**Table 2:** Overlap between the results of this study and published redox proteomes. Overlap between polypeptides found in our study and (**A**) proteins identified in the 2-cysteine peroxiredoxin (2-CysPRX) interactome study of Cerveau et al. [16], (**B**) the ferredoxin (FDX)-thioredoxin (TRX) system [31], and (**C**) the TRX interactome as determined by TRX-trapping in spinach [29]. * = targets bound to 2-CysPRXA in this study.

**(A)**		
**2-CysPRXA Affinity Binding in Arabidopsis**		
**17 of 67 (25%) Overlap with [16]**		
*	Ribulose bisphosphate carboxylase large subunit	O03042
*	Pyruvate dehydrogenase E1; subunit alpha-3	O24457
*	Fructose-1,6-bisphosphatase	P25851
*	Glutathione reductase	P42770
*	3-oxoacyl-[acyl-carrier-protein] synthase 1	P52410
*	Thylakoid lumenal 19 kDa protein	P82658
*	Phospho-2-dehydro-3-deoxyheptonate aldolase 2	Q00218
*	Glutathione S-transferase F8	Q96266
*	2-Cys peroxiredoxin B	Q9C5R8
*	Photosystem II repair protein PSB27-H1	Q9LR64
*	Peroxiredoxin Q	Q9LU86
*	Soluble inorganic pyrophosphatase 6	Q9LXC9
*	50 S ribosomal protein L1	Q9LY66
*	Phosphoglucomutase	Q9SCY0
*	Ferredoxin-dependent glutamate synthase 1	Q9ZNZ7
*	Ferredoxin–NADP reductase 1	F4JZ46
*	Dihydrolipoyl dehydrogenase 2	F4JLP5
**(B)**		
**Proteins Associated with FD/TRX system [31]**		
*	Fructose 1,6-bisphosphatase	
*	Glyceraldehyde 3-P dehydrogenase subunit B	
*	Sedoheptulose 1,7-bis-phosphatase	
*	Phosphoribulokinase	
	Rubisco activase	
*	CP12	
*	NADP-malate dehydrogenase	
	ATP synthase γ-subunit	
	Glucose 6-phosphate dehydrogenase	
*	Acetyl CoA carboxylase	
*	β-Amylase	
*	α-Glucan water dikinase	
*	ADP-glucose pyrophosphorylase	
*	Peptidyl-prolyl cis-trans isomerase	
	2-Cys Peroxiredoxin	
**(C)**		
**TRX-Trapping in Spinach [29]**		
	4-hydroxy-3-methylbut-2-en-1-yl diphosphate synthase	Q9FF59
*	1-deoxy-D-xylulose-5-phosphate reductoisomerase	Q9SP64
	Glutamate-1-semialdehyde aminotransferase	P31593
*	Uroporphyrinogen decarboxylase	Q42967
	Magnesium-chelatase subunit ChlI-1	P16127
	Phosphomethylpyrimidine synthase	O82392
*	Thiamine thiazole synthase	Q38814
*	Heat shock 70 protein	O50036
*	RuBisCO large subunit-binding protein subunit alpha	P08926
	RuBisCO large subunit-binding protein subunit beta	P08927
*	ATP-dependent Clp protease	P31541
*	Carbonic anhydrase	P16016
*	Beta-amylase 1	P16098
	Enolase 1	Q9LEJ0
	ATP-dependent DNA helicase homolog RECG	Q9ZVG0
	LlFtsZ protein	Q9LRC5
	Transketolase	O20250
	Triosephosphate isomerase	P48496
*	Ribulose-phosphate 3-epimerase	Q43157
	D-3-phosphoglycerate dehydrogenase	Q9FSS6
	O-acetylserine (thiol)-lyase B	P32260
	28 kDa ribonucleoprotein	P28644
*	Elongation factor Tu	Q43467
	Elongation factor G-1	P34811
*	30S ribosomal protein S1	P29344
*	30S ribosomal protein S6 alpha	P82403
	6-phosphogluconate dehydrogenase, decarboxylating 2	Q94KU2
*	Sedoheptulose-1,7-bisphosphatase	O20252
*	Phosphoribulokinase	P09559
*	Glyceraldehyde-3-phosphate dehydrogenase B	P12860
	Ribulose bisphosphate carboxylase/oxygenase activase	P10871
*	Ribulose bisphosphate carboxylase small chain 2	Q43832
*	Malate dehydrogenase [NADP]	P52426
*	Glutamate--ammonia ligase	Q9LVI8
*	Biotin carboxylase	O23960
	2Cys-peroxiredoxin	Q9SQJ4

## Supplementary Materials

The following are available online at https://www.mdpi.com/2076-3921/9/6/515/s1, Table S1: Assignment of polypeptides identified as interactors of 2-CyspRX to gene ontology groups; Table S2: Compilation of all 178 polypeptides identified in this study and depiction of the specific conditions for their elution; Table S3: Complete list of all 178 proteins eluted in the pull down assay presented here; Figure S1: Structure of lipoxygenase 2 and analysis of Cys residues; Figure S2: Structure of chorismate synthase (CS) and analysis of Cys residues.
