# Supplementary material for: Redox Conformation-Specific Protein–Protein Interactions of the 2-Cysteine Peroxiredoxin in Arabidopsis
Source: Antioxidants (Basel). 2020 Jun 11;9(6):515. doi: 10.3390/antiox9060515 (PMC7346168; doi:10.3390/antiox9060515)
Supplement: Supplementary file 1 [file antioxidants-09-00515-s001.pdf]

Suppl. Table 1: Liebthal et al.

photosynthesis	31	Co-factor and vitamin metabolism	1
major CHO metabolism	8	tetrapyrrole synthesis	4
minor CHO metabolism	2	stress	8
glycolysis	8	redox	11
OPP	3	nucleotide metabolism	6
TCA / org transformation	5	C1-metabolism	2
mitochondrial electron transport / ATP synthesis	1	misc	8
lipid metabolism	12	RNA	5
N-metabolism	2	protein	28
amino acid metabolism	11	cell	1
metal handling	1	development	1
secondary metabolism	5	transport	1
hormone metabolism	4	not assigned	13

Suppl. Table 1: Functional assignments for all 178 protein targets of 2CysPRX assigned to the chloroplast including 4 double mapped proteins.

Suppl. Table 2: Liebthal et al.

20 kDa chaperonin	At5g20720	Glyceraldehyde-3-phosphate dehydrogenase GAPA1	AT3G26650
2-Cys peroxiredoxin BAS1	At3g11630	Glyceraldehyde-3-phosphate dehydrogenase GAPA2	AT1G12900
2-Cys peroxiredoxin BAS1-like	AT5G06290	Glyceraldehyde-3-phosphate dehydrogenase GAPB	AT1G42970
2-isopropylmalate synthase 1	AT1G18500	Heat shock 70 kDa protein 7	AT5G49910
3-oxoacyl-[acyl-carrier-protein] reductase	AT1G24360	Lectin-like protein At5g03350	AT5G03350
3-oxoacyl-[acyl-carrier-protein] synthase I	AT5G46290	Leucine aminopeptidase 3	AT4G30910
50S ribosomal protein L12-1	AT3G27830	Leucine-rich repeat-containing protein	AT3G20820
Adenylosuccinate lyase	AT4G18440	MLP-like protein 43	AT1G70890
Aspartate aminotransferase 3	AT5G11520	Nucleoside diphosphate kinase III	AT4G11010
Aspartate--tRNA ligase 2	AT4G31180	Oxalate--CoA ligase	AT3G48990
Aspartyl aminopeptidase	AT5G04710	Peptidyl-prolyl cis-trans isomerase	AT2G10940
ATP synthase subunit beta	AtCg00480	Phosphoenolpyruvate carboxylase 2	AT2G42600
Beta carbonic anhydrase 1	AT3G01500	Phosphoribulokinase	AT1G32060
Bifunctional monothiol glutaredoxin-S16	AT2G38270	Pyruvate dehydrogenase E1 component subunit alpha-3	AT1G01090
Cobalamin-independent methionine synthase 2	AT3G03780	Ribulose bisphosphate carboxylase large chain	AtCg00490
Cystathionine beta-lyase	AT3G57050	Ribulose bisphosphate carboxylase small chain 1A	AT1G67090
Dehydrin ERD14	AT1G76180	Ribulose bisphosphate carboxylase small chain 1B	AT5G38430
Diaminopimelate epimerase	AT3G53580	Sedoheptulose-1,7-bisphosphatase	AT3G55800
Dihydrolipoyl dehydrogenase 2	AT4G16155	Serine/threonine protein phosphatase 2A regulatory subunit	AT1G13930
D-ribulose-5-phosphate-3-epimerase	At5g61410	Stearoyl-[acyl-carrier-protein] 9-desaturase 7	AT2G43710
Fructose-1,6-bisphosphatase	AT3G54050	Thioredoxin F1	AT3G02730
Fructose-bisphosphate aldolase 2	AT4G38970	Tryptophan--tRNA ligase	AT2G25840
Fructose-bisphosphate aldolase 8	At3g52930	UDP-sulfoquinovose synthase	AT4G33030
Glucose-6-phosphate 1-dehydrogenase (Fragment)	AT5G35790	Uncharacterized protein	AT4G03200
Glyceraldehyde 3-phosphate dehydrogenase	AT1G13440	Uroporphyrinogen decarboxylase 1	AT3G14930

Suppl. Table 2: Summary of proteins eluted with 2-CysPRXA WT and both amino acid variants (50).

Suppl. Table 3: Liebthal et al.

Protein name	gene	2-CysPRXA WT					pseudo-reduced C54S					pseudo-hyperoxidized C54D				
		R-S	R-I	O-S	O-D	O-I	R-S	R-I	O-S	O-D	O-I	R-S	R-I	O-S	O-D	O-I
1-deoxy-D-xylulose 5-phosphate reductoisomerase	AT5G62790	X					X									
20 kDa chaperonin	At5g20720	X					X							X		
2-C-methyl-D-erythritol 2,4-cyclodiphosphate synthase	AT1G63970								X							
2-Cys peroxiredoxin BAS1	At3g11630				X					X					X	
2-Cys peroxiredoxin BAS1-like	AT5G06290		X		X	X		X	X		X					
2-isopropylmalate synthase 1	AT1G18500		X								X				X	
30S ribosomal protein S10	AT3G13120										X					
30S ribosomal protein S17	AT1G79850	X														
30S ribosomal protein S3	ATCG00800			X		X										
30S ribosomal protein S6 alpha	AT1G64510						X									
30S ribosomal protein S9	AT1G74970	X					X									
3-oxoacyl-[acyl-carrier-protein] reductase	AT1G24360	X					X					X		X		
3-oxoacyl-[acyl-carrier-protein] synthase I	AT5G46290		X			X	X	X			X					X
4-diphosphocytidyl-2-C-methyl-D-erythritol kinase	AT2G26930	X														
4-hydroxy-tetrahydrodipicolinate reductase 1	AT2G44040								X							
4-hydroxy-tetrahydrodipicolinate synthase 1	AT3G60880													X		
4-hydroxy-tetrahydrodipicolinate synthase 2	AT2G45440			X												
50S ribosomal protein L1	AT3G63490	X										X				
50S ribosomal protein L11	AT1G32990			X										X		
50S ribosomal protein L12-1	AT3G27830	X					X	X						X		
50S ribosomal protein L27	AT5G40950								X							
50S ribosomal protein L29	AT5G65220	X														
50S ribosomal protein L31	AT1G75350	X					X									
50S ribosomal protein L5	AT4G01310								X							
50S ribosomal protein L6	AT1G05190	X					X									
60S ribosomal protein L26-1	AT3G49910	X														
Acyl-acyl carrier protein thioesterase ATL3	AT1G68260											X				
Adenylosuccinate lyase	AT4G18440		X	X			X	X	X	X	X	X			X	X
Adenylosuccinate synthetase	AT3G57610													X		
Alanine--tRNA ligase	AT5G22800			X										X		
Allene oxide synthase	AT5G42650													X		
Alpha-glucan water dikinase 1	AT1G10760	X					X									
Alpha-xylosidase 1	AT1G68560								X			X		X		
Arginase 2	AT4G08870							X								
Aspartate aminotransferase 3	AT5G11520	X					X					X				
Aspartate--tRNA ligase 2	AT4G31180	X					X							X		
Aspartyl aminopeptidase	AT5G04710	X					X					X			X	
AT3g28220/T19D11_3	AT3G28220						X									
AT5g64380/MSJ1_22	AT5G64380													X		
ATP synthase subunit beta	AtCg00480	X					X		X					X		
ATPase alpha subunit (Fragment)	AtCg00120								X							

		2-CysPRXA WT					pseudo-reduced C54S					pseudo-hyperoxidized C54D				
Protein name	gene	R-S	R-I	O-S	O-D	O-I	R-S	R-I	O-S	O-D	O-I	R-S	R-I	O-S	O-D	O-I
ATPase, F1 complex, alpha subunit protein	AT2G07698	X								X						
ATP-dependent 6-phosphofructokinase 5	AT2G22480														X	
Beta carbonic anhydrase 1	AT3G01500	X		X			X							X		
Beta carbonic anhydrase 2	AT5G14740							X			X					
Beta-amylase 2	AT4G00490	X		X												
Beta-amylase 3	AT4G17090										X					
Bifunctional monothiol glutaredoxin-S16	AT2G38270	X					X		X			X				
Bifunctional protein FolD 2	AT3G12290														X	
Biotin carboxyl carrier protein of acetyl-CoA carboxylase 1	AT5G16390							X								
Biotin carboxylase	AT5G35360							X								
Calvin cycle protein CP12-1	AT2G47400								X					X		
Chaperone protein ClpC1	AT5G50920		X					X								
Chaperonin 60 subunit alpha 1	AT2G28000		X					X								
Chaperonin 60 subunit beta 3	AT5G56500	X										X				
Chlorophyll a-b binding protein 2	AT1G29920	X														
Chloroplast stem-loop binding protein of 41 kDa b	AT1G09340									X					X	
Chorismate synthase	AT1G48850	X														
Cobalamin-independent methionine synthase 2	AT3G03780			X			X						X			
Cystathionine beta-lyase	AT3G57050					X					X				X	
Dehydrin ERD14	AT1G76180	X					X	X				X				
Delta-aminolevulinic acid dehydratase 1	AT1G69740	X														
Diaminopimelate epimerase	AT3G53580				X					X					X	
Dihydrolipoyl dehydrogenase 2	AT4G16155	X					X					X				
Dihydroxy-acid dehydratase	AT3G23940													X		
D-ribulose-5-phosphate-3-epimerase	At5g61410	X					X					X				
Early nodulin-like protein 2	AT4G27520	X					X									
Elongation factor 1 alpha (Fragment)	At4g20360			X												
E-Z type HEAT repeat-containing protein	AT3G62530													X		
Ferredoxin-dependent glutamate synthase 1	AT5G04140							X								
Ferredoxin--NADP reductase, leaf isozyme 1	AT5G66190	X					X									
Ferredoxin--NADP reductase, leaf isozyme 2	AT1G20020						X									
Ferritin-4	AT2G40300													X	X	
FGGY family of carbohydrate kinase	AT4G30310				X											
Formate--tetrahydrofolate ligase	AT1G50480									X						
Fructose-1,6-bisphosphatase	AT3G54050	X		X					X			X		X		
Fructose-bisphosphate aldolase 1	At2g21330									X					X	
Fructose-bisphosphate aldolase 2	AT4G38970				X					X					X	
Fructose-bisphosphate aldolase 5	At4g26530							X	X							
Fructose-bisphosphate aldolase 8	At3g52930	X							X					X		
GAN	At1g55480						X									
GDSL esterase/lipase ESM1	AT3G14210							X								

Protein name	gene	2-CysPRXA WT					pseudo-reduced C54S					pseudo-hyperoxidized C54D				
		R-S	R-I	O-S	O-D	O-I	R-S	R-I	O-S	O-D	O-I	R-S	R-I	O-S	O-D	O-I
GEM-like protein 1	AT1G28200								X					X		
Glucose-1-phosphate adenylyltransferase large subunit 1	AT5G19220		X						X							
Glucose-1-phosphate adenylyltransferase small subunit	AT5G48300		X					X	X							
Glucose-6-phosphate 1-dehydrogenase (Fragment)	AT5G35790	X		X				X						X		
Glutamine synthetase cytosolic isozyme 1-3	AT5G35630							X			X					
Glutamyl-tRNA(Gln) amidotransferase subunit A	AT3G25660	X					X									
Glutathione reductase	AT3G54660													X		
Glutathione S-transferase F8	AT2G47730						X									
Glyceraldehyde 3-phosphate dehydrogenase	AT1G13440				X	X				X	X				X	X
Glyceraldehyde-3-phosphate dehydrogenase GAPA1	AT3G26650		X		X			X		X			X		X	
Glyceraldehyde-3-phosphate dehydrogenase GAPA2	AT1G12900	X	X		X	X	X	X					X			
Glyceraldehyde-3-phosphate dehydrogenase GAPB	AT1G42970				X					X					X	
Glycine--tRNA ligase, chloroplastic/mitochondrial 2	AT3G48110						X		X							
HAD-superfamily hydrolase, subfamily IG, 5'-nucleotidase	AT5G48960													X		
Heat shock 70 kDa protein 7	AT5G49910		X		X			X							X	
Isoamylase 3	AT4G09020										X					
Lectin-like protein At5g03350	AT5G03350		X			X		X					X			
Leucine aminopeptidase 3	AT4G30910			X					X					X		
Leucine-rich repeat-containing protein	AT3G20820	X		X			X		X			X				
Lipoxygenase 2	AT3G45140	X					X									
Malate dehydrogenase	AT3G47520	X														
MLP-like protein 423	AT1G24020	X					X									
MLP-like protein 43	AT1G70890				X					X					X	
Monodehydroascorbate reductase 6	At1g63940								X							
Myrosinase 2	AT5G25980							X								
NADP-dependent malic enzyme 2	AT5G11670									X						
NADPH-dependent thioredoxin reductase 3	AT2G41680				X											
Nucleoside diphosphate kinase III	AT4G11010				X					X					X	
Oxalate--CoA ligase	AT3G48990			X					X			X		X		
Oxygen-evolving enhancer protein 1-1	AT5G66570									X						
Oxygen-evolving enhancer protein 1-2	AT3G50820		X					X								
Oxygen-evolving enhancer protein 3-1	AT4G21280							X					X		X	
Peptidyl-prolyl cis-trans isomerase	AT2G10940		X				X	X				X				
Peptidyl-prolyl cis-trans isomerase CYP20-3	AT3G62030													X		
Peroxiredoxin Q	AT3G26060														X	
Peroxiredoxin-2E	AT3G52960								X					X		
Phospho-2-dehydro-3-deoxyheptonate aldolase 1	At4g39980	X														
Phospho-2-dehydro-3-deoxyheptonate aldolase 2	AT4G33510					X		X								
Phosphoenolpyruvate carboxylase 2	AT2G42600					X		X		X					X	
Phosphoglucan phosphatase LSF1	AT3G01510	X														
Phosphoglucan, water dikinase	AT5G26570												X			

Protein name	gene	2-CysPRXA WT					pseudo-reduced C54S					pseudo-hyperoxidized C54D				
		R-S	R-I	O-S	O-D	O-I	R-S	R-I	O-S	O-D	O-I	R-S	R-I	O-S	O-D	O-I
Phosphoglucomutase	AT5G51820	X					X									
Phosphoglycerate kinase 2	AT1G56190						X		X					X	X	
Phospholipid hydroperoxide glutathione peroxidase 1	AT2G25080						X		X					X		
Phosphoribosylaminoimidazole carboxylase like protein	AT2G37690							X								
Phosphoribulokinase	AT1G32060					X		X			X		X			
Photosystem I reaction center subunit II-1	AT4G02770						X									
Photosystem I reaction center subunit IV B	AT2G20260								X					X		
Photosystem II repair protein PSB27-H1	AT1G03600	X												X		
Photosystem II stability/assembly factor HCF136	AT5G23120													X		
Plastidial pyruvate kinase 1	AT3G22960			X										X		
Plastidial pyruvate kinase 2	AT5G52920														X	
Plastidial pyruvate kinase 3	AT1G32440													X		
Plastocyanin major isoform	AT1G20340													X		
PLAT domain-containing protein 1	AT4G39730	X														
Porphobilinogen deaminase	AT5G08280									X					X	
Probable protein phosphatase 2C 62	AT4G33500	X														
Protease Do-like 1	AT3G27925						X									
Protein COLD-REGULATED 15A	AT2G42540						X									
Protein COLD-REGULATED 15B	AT2G42530						X									
Putative uncharacterized protein	At5g24490										X			X		
Putative uncharacterized protein	At2g22230						X									
Putative uncharacterized protein At3g52150	At3g52150	X					X									
Pyruvate dehydrogenase E1 component subunit alpha-3	AT1G01090	X					X		X			X				
Pyruvate dehydrogenase E1 component subunit beta-3	AT2G34590	X														
Red chlorophyll catabolite reductase	AT4G37000													X		
Ribose-phosphate pyrophosphokinase 3	AT1G10700													X		
Ribulose bisphosphate carboxylase large chain	AtCg00490					X					X					X
Ribulose bisphosphate carboxylase small chain 1A	AT1G67090		X		X			X			X				X	
Ribulose bisphosphate carboxylase small chain 1B	AT5G38430				X			X		X					X	
RNA recognition motif-containing protein	AT4G09040	X					X									
RNA-binding protein CP29B, chloroplastic	AT2G37220									X					X	
Sedoheptulose-1,7-bisphosphatase	AT3G55800			X					X					X		
Serine/threonine protein phosphatase 2A regulatory subunit	AT1G13930	X					X					X		X		
Short-chain dehydrogenase/reductase family protein	At4g20760											X		X		
Single-stranded DNA-binding protein WHY1	AT1G14410	X														
Single-stranded DNA-binding protein WHY3	AT2G02740	X					X									
Soluble inorganic pyrophosphatase 6	AT5G09650						X									
SpoU_methylase domain-containing protein	AT5G14910	X					X									
Starch synthase 3	AT1G11720													X		
Stearoyl-[acyl-carrier-protein] 9-desaturase 5	AT3G02630													X		
Stearoyl-[acyl-carrier-protein] 9-desaturase 7	AT2G43710	X					X							X		

		2-CysPRXA WT					pseudo-reduced C54S					pseudo-hyperoxidized C54D				
Protein name	gene	R-S	R-I	O-S	O-D	O-I	R-S	R-I	O-S	O-D	O-I	R-S	R-I	O-S	O-D	O-I
Sulfiredoxin	AT1G31170		X													
Thiamine thiazole synthase	AT5G54770													X		
Thioredoxin F1	AT3G02730	X					X					X		X		
Thioredoxin M1	AT1G03680						X						X			
Thioredoxin M4	AT3G15360						X						X			
Thiosulfate sulfurtransferase 16	AT5G66040													X		
Thylakoid lumenal 19 kDa protein	AT3G63540														X	
Transaldolase-like protein	AT1G12230	X														
Transketolase-1	AT3G60750		X													
Tryptophan--tRNA ligase	AT2G25840			X			X		X					X		
UDP-sulfoquinovose synthase	AT4G33030				X					X					X	
Uncharacterized protein 1	AT4G03200	X					X								X	
Uncharacterized protein 2	AT5G40450								X					X		
Uncharacterized protein 3	At2g27680	X														
Uroporphyrinogen decarboxylase 1	AT3G14930	X					X		X	X					X	

Suppl. Table 3: Complete list of all 178 proteins eluted in the pull down assay presented here. Proteins are given in alphabetical order with their corresponding gene. Colors are related to Fig. 3 for group assignments. Amino acid variants are set as main groups with their downstream redox treatments (R: reduced; O: oxidized) and elutions (S: salt, NaCl; D: DTT; I: Imidazole).

Suppl. Fig. 1: Liebthal et al.

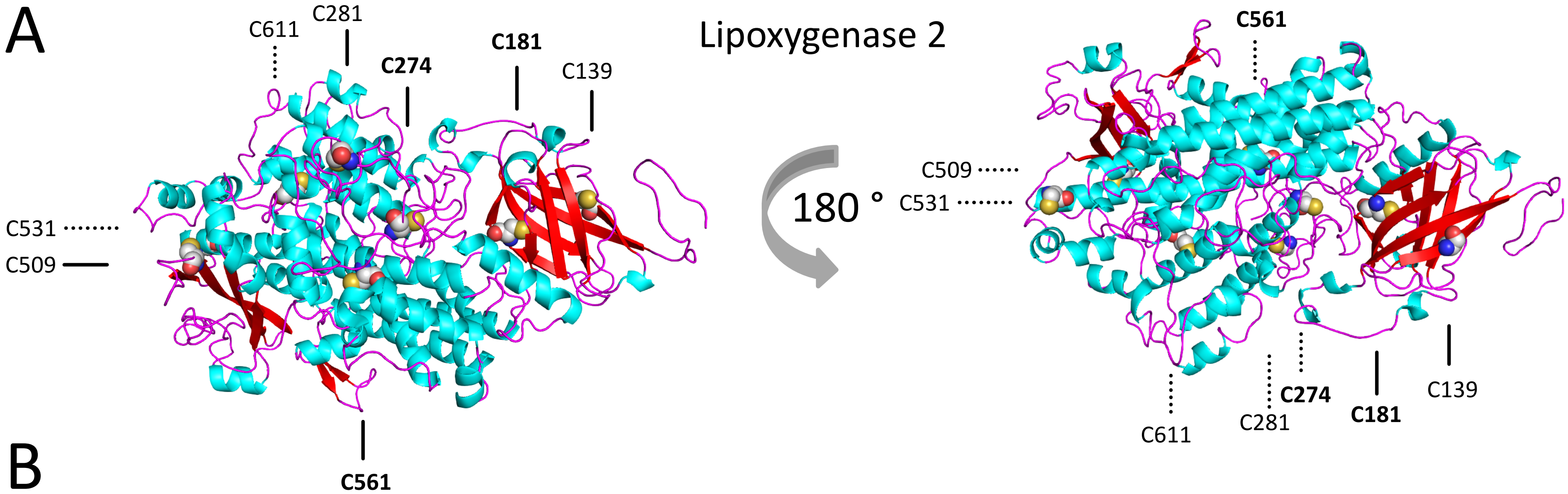

B

UniProtID
P38418 (LOX2_ARATH)

Seq Length
896

Homologues
14

Putative conserved
3 of 8

D7LNL7_ARALL
E1Z2P2_CHLVA
A9SEG6_PHYPA
A0A194YND7_SORBI
Q06XS2_MAIZE
A0A2K3D2I6_CHLRE
F6HB91_VITVI
A0A2K1Z1H6_POPTR
I0YJS7_COCSC
D8SDE9_SELML
Q6RSN2_CARPA
Q6H7Q6_ORYSJ
B8AJB1_ORYSI
A4S026_OSTLU

Cys	Position	Score	P-Value
Cys 1	3 (TP region)	0.071429	0.999891
Cys 2	50 (TP region)	0.071429	0.999891
Cys 3	139	0.071429	0.999891
Cys 4	181	0.714286	0.041224
Cys 5	274	0.642857	0.122727
Cys 6	281	0.142857	0.998378
Cys 7	509	0.071429	0.999891
Cys 8	531	0.214286	0.988874
Cys 9	561	0.714286	0.041224
Cys 10	611	0.285714	0.953032

Suppl. Fig. 1: Structural model and cysteinyl residues of LIPOXYGENASE 2 (LOX2) (A) The protein structure of AtLOX2 (LOX2_ARATH, without transit peptide) was generated with the RaptorX prediction tool and further modeled in Pymol. The structures on the left and right hand side were rotated by 180° upside down. (B) Phylogenetic analysis of the Cys residues of LOX2 of *Arabidopsis thaliana*. Each Cys residue was analyzed with the tool ConCysFind. Three out of 10 Cys revealed a high degree of phylogenetic conservation as indicated by Cys scores >0.5 (labeled in green) and a low p-value. Reference: Kallberg, M.; Wang, H.; Wang, S.; Peng, J.; Wang, Z.; Lu, H.; Xu, J. Template-based protein structure modeling using the RaptorX web server. *Nature Protocols* **2012**, *7*, 1511-1522.

Suppl. Fig. 2: Liebthal et al.

A

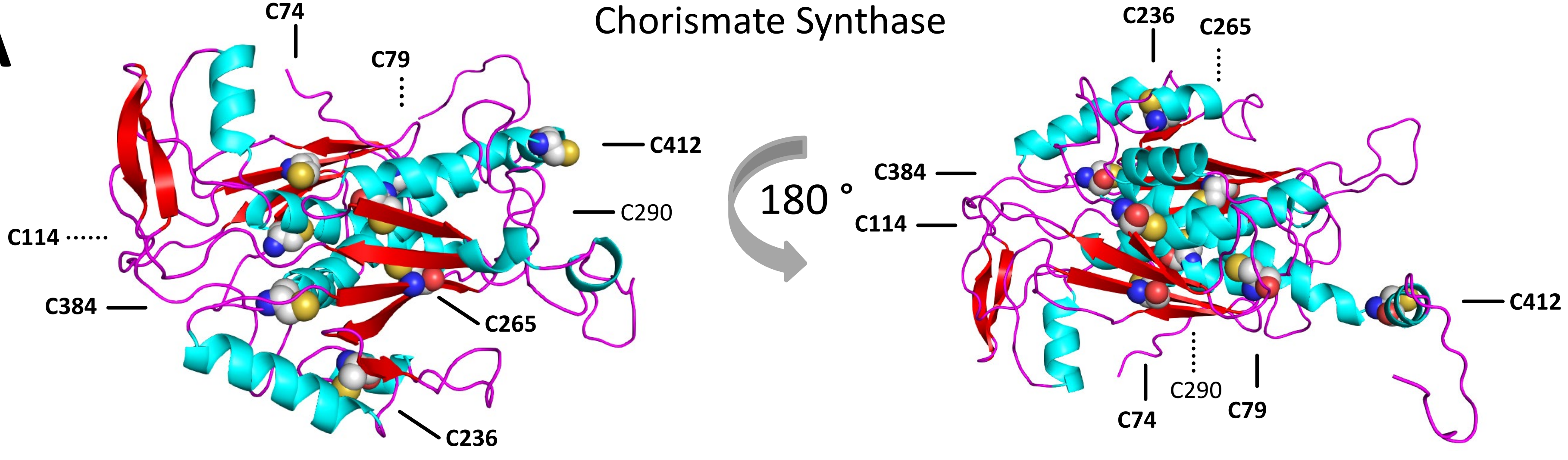

B

**UniProtID**	**Seq Length**
P57720_ARATH	436
**Homologues**	**Putative conserved**
18	7 of 8

D7KD60_ARALL
E1ZRW2_CHLVA
I0Z006_COCSC
A0A2K3DVB7_CHLRE
A9RTV8_PHYPA
D8RQP2_SELML
A0A090M7W8_OSTTA
A9PGP7_POPTR
D7TMA3_VITVI
M2W447_GALSU
B4FLA2_MAIZE
C5WQV1_SORBI
M1VFJ1_CYAME
B8AKA5_ORYSI
Q10NY1_ORYSJ
A4RTB7_OSTLU
C1E5R8_MICCC

**Cys 1 Position**	**Cys 1 Score**	**Cys 1 P-Value**
74	0.888889	0.000001
**Cys 2 Position**	**Cys 2 Score**	**Cys 2 P-Value**
79	0.611111	0.020000
**Cys 3 Position**	**Cys 3 Score**	**Cys 3 P-Value**
114	0.833333	0.000020
**Cys 4 Position**	**Cys 4 Score**	**Cys 4 P-Value**
236	1.000000	4.894E-10
**Cys 5 Position**	**Cys 5 Score**	**Cys 5 P-Value**
265	1.000000	4.894E-10
**Cys 6 Position**	**Cys 6 Score**	**Cys 6 P-Value**
290	0.111111	0.996391
**Cys 7 Position**	**Cys 7 Score**	**Cys 7 P-Value**
384	1.000000	4.894E-10
**Cys 8 Position**	**Cys 8 Score**	**Cys 8 P-Value**
412	0.944444	5.548E-8

Suppl. Figure 2: Protein structure of AtCS and conserved cysteines. (A) Structures were processed using the AROC_ARATH sequence excluding transit peptide in RaptorX prediction tool and further modelled in Pymol. Conserved Cys residues were analyzed in ConCysFind and are highlighted in bold. 3D-dependent distortion of Cys locations is indicated with a dashed line aiming for Cys behind or inside the polpeptide. (B) ConCysFind summary of AROC_ARATH sequence with default settings for thresholds and scores. Homologue UniProt IDs are listed for 18 plant species. Cys are given with an amino acid position and ascending order from N- to C-terminal ends. Score and p-values reflect conservation for 7 out of 8 Cys. With several neighboring and solvent accessible Cys, a high probability for disulfide formation and redox regulation present.
